# Comprehensive lipidomics analysis reveals the changes in lipid profile of camellia oil affected by insect damage

**DOI:** 10.3389/fnut.2022.993334

**Published:** 2022-09-02

**Authors:** Qingyang Li, Wei Zhang, Danyu Shen, Zhihong Li, Jinping Shu, Yihua Liu

**Affiliations:** Research Institute of Subtropical Forestry, Chinese Academy of Forestry, Fuyang, China

**Keywords:** camellia oil, lipidomic, lipid profile, insets damage, biotic stress, nutrition

## Abstract

Information on changes in lipid composition of seed oils under biotic stresses is scare. The camellia weevil, Curculio chinensis (Coleoptera: Curculionidae) as a notorious seed predator of Camellia species, has caused huge economic losses in China. Lipidomics is used in this study to reveal the lipid composition of camellia oil and its changes after insect damage. 278 lipids including glycerolipids (GL) (221), glycerophospholipids (GP) (34), fatty acyls (FA) (13), sphingolipids (SP) (8), prenol lipids (PR) (1) and sterol lipids (ST) (1) were determined in camellia oils. Insect damage had a significant impact on lipids, particularly FA and GL. Ten significantly different lipids [FFA(18:2), FFA(24:6), TG(14:1/18:2/18:2), TG(16:0/23:0/18:2), TG(20:1/24:1/18:2), TG(18:2/24:0/18:2), TG(16:3/18:2/22:5), PI(16:1/18:1), PE(16:0/18:1), PE(18:1/18:2)] were identified as potential biomarkers for distinguishing oil extracted from non-infested oilseeds and oil from infested oilseeds. We also detected four most important metabolic pathways by bioinformatics analysis to explore the mechanisms underlying changes. Our findings may be useful for future camellia oil production and may provide new insight into improving of nutritional quality of camellia oil.

## Introduction

Seed oils are rich in a variety of nutrients including dietary fats and phytochemicals, and they have medical and commercial applications (1, 2). Lipids are the most abundant component of seed oils, and their composition has a direct impact on the nutritional quality of the seed oil, which is critical to human health (3, 4). In the past, the main focus of seed oil lipid studies has been on oil yield and the fatty acid composition of the extracted lipids (5). However, fatty acids analysis does not provide information about the matrix’s total lipid content (6). It is thus to obtain more detailed information on the composition and chemical structures of these lipids. Lipidomics is a powerful tool that uses mass spectrometry to reveal information about the distribution of intact the profile of lipid molecules in biological samples (7, 8). Recently, lipidomic has been used in food science for complex lipid analysis, such as quality assessment (9–11), quality identification (12) and origin traceability (7). In addition, as an emerging technology, lipidomics has been applied to the lipid exploration of various oils, including peanut oil (13, 14), hazelnut oil (15, 16), and flaxseed oil (17). However, successful application of lipidomic analysis in characterization of lipid profile in edible oil is very limited (17). Based on lipidomics, several researchers have also investigated the effects of abiotic stresses (such as light and temperature) on oil lipids (17, 18). The distribution of lipid classes in rapeseed oil changed after 12 days of light storage, with a decrease in triacylglycerol and an increase in diacylglycerol and phospholipids (18). After 24 days of accelerated storage, 51 significantly different lipids in hazelnut oil were screened, and these lipids could be used as biomarkers to detect the change of the metabolic pathways in hazelnut seeds during storage (15). Unfortunately, unlike the limited lipidomic studies on abiotic stresses of oil/oilseed, the literature on lipid changes in oil/oilseed subjected to biotic stresses is limited, particularly in lipidomic analysis for seeds stressed by insect damage.

**FIGURE 1:**
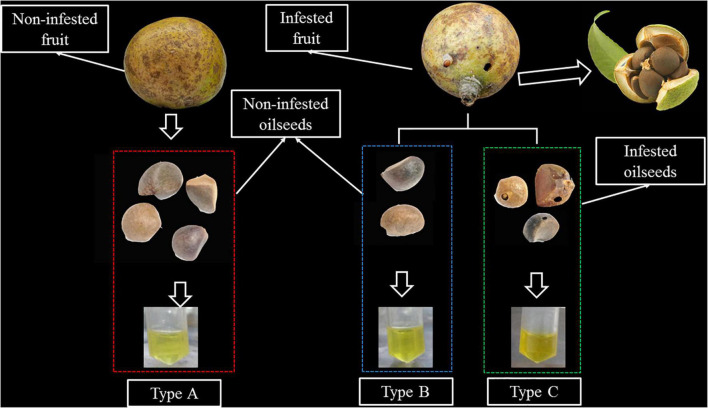
An illustration of the different types of samples. Type A: Oil extracted from non-infested oilseeds of non-infested fruit. Type B: oil extracted from non-infested oilseeds of Curculio chinensis Cheveolat infested fruit. Type C: oil extracted from infested oilseeds of Curculio chinensis Cheveolat infested fruit.

Camellia oil is an edible oil obtained from camellia oleifera seeds, which is widely cultivated in China, accounting for about 90% of the global total production (5). Camellia oil is not only a good source of nutrients, but also a food source with health benefits. These advantages are due to the numerous phytonutrients and fatty acid profiles found in them (5). The camellia weevil, Curculio chinensis Cheveolat (Coleoptera: Curculionidae), is a well-known seed predator of Camellia trees, including Camellia oleifera and Camellia. Sinensis which are widely cultivated around the world. Adult camellia weevils emerge from soil in late April to early May and lay eggs in the seeds primarily in June. Larvae spend 3 months living in and feeding on seeds before emerging from the seed and overwintering in the soil (19). The larval feeding causes significant quality and yield losses to seeds (19–21). To the best of our knowledge, no study has yet identified changes in the lipidome and related metabolic pathways in camellia oil affected by insects. More importantly, unlike previous reports on oil fruit containing only one oilseed, oil tea fruit contains multiple oilseeds (2–20) in a single fruit, implying that infested and non-infested oilseeds will coexist in the same fruit. However, no previous research has been conducted on the differences in lipid composition of non-infested oilseeds from infested fruit and non-infested fruit. Thus, three types of oils were investigated in this study: Type A (oil extracted from non-infested oilseeds of non-infested fruit), Type B (oil extracted from non-infested oilseeds of C. cheveolat infested fruit), and Type C (oil extracted from infested oilseeds of C. cheveolat infested fruit) (details could be seen in Figure 1). The objective of this study was to (1) compare the lipidomic profiles of camellia oils from camellia oleifera seeds that were not or were infested with camellia weevil; (2) evaluate the effect of biotic stress on camellia oil; and (3) characterize changes in the lipidome and related metabolic pathways of three types of oils.

## Materials and methods

### Chemicals and reagents

HPLC-grade acetonitrile(ACN), methanol (MeOH), isopropanol (IPA), dichloromethane (CH_2_Cl_2_), methyl tert-butyl ether (MTBE) were purchased from Merck (Darmstadt, Germany). HPLC-grade formic acid (FA), Ammonium for mate (AmFA) were purchased from Sigma-Aldrich (St. Louis, MO, United States). Ultrapure water was obtained by a Milli-Q system (Millipore, Billerica, MA). Lipid standards were purchased from Sigma-Aldrich or Avanti Polar Lipids (Alabaster, AL).

### Preparation of camellia oil samples

The experimental materials for this study collected from the main cultivars of the Changlin series of camellia oilseeds in Zhejiang Province, China. All samples came from 9- to 11-year-old trees of local orchard during mature stage. Approximately 2 kg Camellia fruits with the same degree of cracking (to ensure same degree of maturity) are randomly collected from each collection location. Camellia oilseeds are classified into three types *via* visual observation of the occurrence of insect digged holes on the fruit shell and seed shell of camellia (Figure 1). The fruit shell and seed shell were manually cracked. It is then dried in an oven at 60°C until it reaches a constant mass, after which it is ground to obtain finely pulverized material. Petroleum ether was used as an organic solvent to obtain the oils by means of Soxhlet extraction. Camellia oils are classified into three types according to the occurrence of the camellia weevil damage: Type A: oil extracted from non-infested oilseeds of non-infested fruit; Type B: oil extracted from non-infested oilseeds of infested fruit; Type C: oil extracted from infested oilseeds. The extracted oil was packed in an amber bottle and kept in −20°C till analysis.

### Lipid extraction procedure

Sample of 10 mg was added to the 2 mL centrifugal tube. Add 1 mL of a mixture of ACN and IPA (ACN: IPA = 1:1, V/V) to re-dissolve and vortexed for 1 min. 10 μL of the previous diluent was added 20 μL of 10 μM internal standard mixed working solution and 970 μL of lipid reconstituted solution, after that shaken for 1 min and centrifuged at 12,000 r/min at 4°C for 3 min. 120 μL Supernatant solution was collected for LC-MS/MS analysis.

### Instruments and methods

The widely targeted lipidomic profiling was performed by MetWare (Wuhan, China) using an LC-ESI-MS/MS system (UPLC, ExionLC AD^1^; MS, QTRAP^®^System^2^). The analytical conditions were as follows:

UPLC Conditions. Column: Thermo Accucore™C30 (2.6 μm, 2.1 mm × 100 mm i.d.); solvent system, A: ACN/water (60/40,V/V, 0.1% formic acid, 10 mmol/L ammonium formate), B: ACN /IPA (10/90VV/V, 0.1% formic acid, 10 mmol/L ammonium formate); Gradient elution program: 80:20 (V/V) for A/B at 0 min, 70:30 (V/V) at 2 min, 40:60 (V/V) at 4 min, 15:85 (V/V) at 9 min, 10:90 (V/V) at 14 min, 5:95 (V/V) at 15.5 min, 5:95 (V/V) at 17.3 min, and 80:20 (V/V) at 17.5 min and 80:20 (V/V) at 20 min; The flow rate was 0.35 ml/min; The column temperature was 45°C; injection volume 2 μL. The effluent was alternatively connected to an ESI-triple quadrupole-linear iontrap (QTRAP)-MS.

ESI-MS/MS Conditions. LIT and triple quadrupole (QQQ) scans were acquired on a triple quadrupole-linear ion trap mass spectrometer (QTRAP), QTRAP^®^ 6,500 + LC-MS/MS System, equipped with an ESI Turbo Ion-Spray interface, operating in positive and negative ion mode and controlled by Analyst 1.6.3 software (Sciex). The ESI source operation parameters were as follows: ion source, turbo spray; source temperature 500°C; ion spray voltage (IS) 5,500 V (Positive), -4,500 V(Negative); Ion source gas 1 (GS1), gas 2 (GS2), curtain gas (CUR) were set at 45, 55, and 35 psi, respectively. Instrument tuning and mass calibration were performed with 10 and 100 μmol/L polypropylene glycol solutions in QQQ and LIT modes, respectively. QQQ scans were acquired as MRM experiments with collision gas (nitrogen) set to 5 psi. DP and CE for individual MRM transitions was done with further DP and CE optimization. A specific set of MRM transitions were monitored for each period according to the metabolites eluted within this period.

### Lipid identification

Qualitative analysis was performed with built-in Metware database (MWDB)^3^ with retention time and ion pairs (Supplementary Figure 1). The MWDB was constructed based on the information available on standard products. In the targeted lipidomics workflow, a previously established MRM method was used to target a large set of ˜1,500 lipids using the SCIEX platform (22). The targeted analysis was accomplished using a “global MRM list” of ˜1,500 lipids which constitute the most commonly identified lipids in plant from 19 different classes of lipids. Quantification was performed in the multiple reaction monitoring (MRM) mode (Supplementary Table 1). Only specified ions were collected. These lipids are analyzed using both positive and negative ion mode in the same run using the fast polarity switching mode of the QTRAP 6,500 +. All the lipids were then quantified by using internal standard lipids (Supplementary Table 2).

### Quality assurance

The camellia oil samples were mixed in equal amounts to obtain a quality control sample (QC sample). Every 10 samples to be analyzed were separated by one QC sample for the duration of the detection to monitor repeatability during the analysis. The high overlaps of the total ion flow of the QC samples, that is, the retention time and peak strength are consistent, indicates that the signal stability of the mass spectrum is good at different times (details could be seen in Supplementary Figure 2).

### Statistical analysis

One-way analysis of variance (ANOVA) was used to test the significance of differences in the lipid content among three types of camellia oils by SPSS22.0. Unsupervised principal component analysis (PCA) was performed by statistics function prcomp within R3.5.1. The orthogonal partial least-squares discriminant analysis (OPLS-DA) was used to discriminate metabolomic profiles between different groups. The variable importance in the projection (VIP) and absolute Log2FC (foldchange) were used to determine the metabolites that were significantly regulated among the groups. The VIP values are extracted from the OPLS-DA results, along with data visualization also with R software.

## Results

### Lipid profiles of camellia oil

**FIGURE 2:**
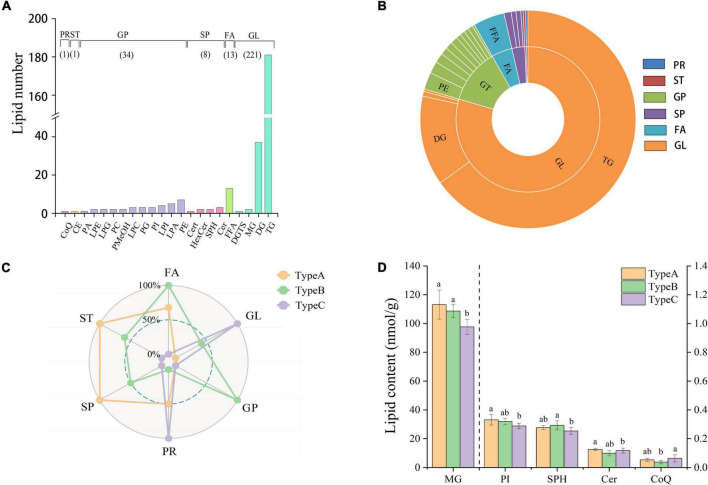
Lipid profile and contents of camellia oil. **(A)** The number of individual lipids in camellia oil from different categories and subclasses. **(B)** Percentage of each lipid categories and subclasses. **(C)** The difference comparison in lipid concentration among the six lipid categories from the three types of oils. **(D)** The difference comparison in lipid concentration among the lipid subclasses from the three types of oils.

A large number of 1,500 lipids were targeted on the SCIEX platform based on previously established MRM methods (22). The results showed that camellia oil samples contained a total of 278 lipids. Lipids were classified into 6 categories [glycerolipids (GL), glycerophospholipids (GP), fatty acyls (FA), sphingolipids (SP), prenol lipids (PR) and sterol lipids (ST)] and 22 subclasses (Figure 2A). As shown in Figure 2B, the number of lipid molecules in different subclasses varied greatly. Notably, GL had the highest number of lipid molecules found, with a proportion of up to 79% of the total amount. There were 221 different types of GLs found, with triacylglycerol (TG) being the most commonly found lipid category (with 181 different types), and diacylglycerol (DG) being the second most commonly found (with 37 different types). In addition, The second highest number of lipid molecules found in GP, with 12.3% of the total amount. There were 34 different types of GPs, including 1 phosphatidic acid (PA), 2 phosphatidylcholines (PCs), 3 phosphatidylglycerols (PGs), 3 phosphatidylinositols (PIs), 7 phosphatidylethanolamines (PEs), 2 lysophosphatidylethanolamines (LPEs), 2 lysophosphatidylglycerols (LPGs), 2 phosphatidyl methanols (PMeOHs), 3 lysophosphatidylcholines (LPCs), 4 lysophosphatidylinositols (LPIs), and 5 lysophosphatidic acids (LPAs). The remaining four lipid classes, with 13 FAs, 8 SPs, 1 ST and 1 PR species, accounted for less than 10% of the total.

### Effect of insect damage on the content of lipid classes of camellia oil

Radar plots reveal the quantitative differences in lipid categories between the three types of oils (Figure 2C). In the lipid categories FA, ST, and SP, we can clearly see that both types of oils extracted from non-infested oilseeds (Type A and Type B) have higher abundance than oil extracted from infested oilseeds (Type C). Furthermore, oil extracted from non-infested oilseeds of the infested fruit (Type B) had a significant advantage in terms of GP, with up to 17.36 nmol/g compared to only 16.81 nmol/g in both Type A and Type C. On the other hand, Type C was high in PR and GL, with contents of 4721.36 and 0.06 nmol/g, respectively. And PR concentration was significantly (*p* < 0.05) higher than that in Type B concentration of 0.04 nmol/g. In terms of lipid subclass, no significant (*p* > 0.05) differences were observed for the majority of them. Only the monoacylglyceride (MG), PI, sphingosine (SPH), ceramide (Cer), and coenzyme Q10 (CoQ) showed significant (*p* < 0.05) differences (Figure 2D). The MG and PI content of Type A was 111.12 and 0.33 nmol/g, respectively, which was 15.88 and 14.85% higher than that of Type C. In addition, the SPH content of Type B was 0.29 nmol/g, which was higher than that in the content of Type C. The content of CoQ in Type C was 0.06 nmol/g, which was 1.7 times higher than the content of CoQ in Type B. Although the majority of differences were found between oil samples extracted from the infested oilseeds and non-infested oilseeds, one lipid subclass (Cer) showed significant (*p* < 0.05) differences between the oil extracted from the two types of non-infested oilseeds. When it comes to the difference of individual lipids, the GP, GL, and FA categories have the largest variation. FFA(24:6), TG(16:0/20:3/20:5), and PE(16:0/18:1) were the top three individual lipids in terms of variation between Type A and Type C, with variation of 76.15, 68.31, and 67.83%, respectively.

### Effect of insect damage on the lipid chain-length of camellia oil

**FIGURE 3:**
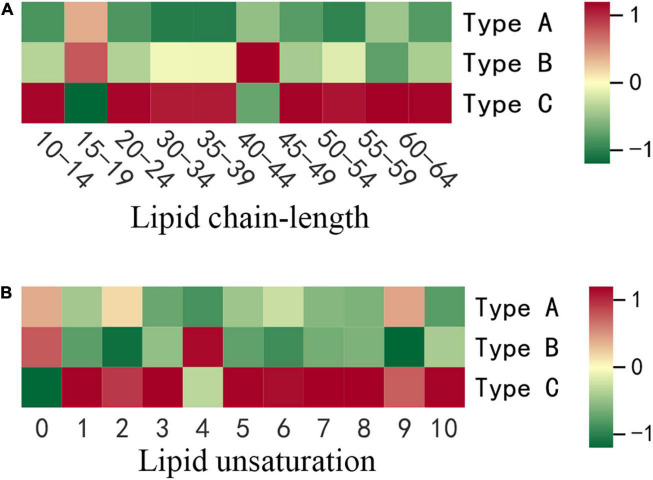
The differences in lipid chain-length and unsaturation content of three types of oils. **(A)** Lipid chain-length. **(B)** Lipid unsaturation. Each colored cell on the map corresponds to concentration of different lipid species. Red color indicates high and green color indicates low.

The sum of the carbon atoms in the fatty acid chains in the lipid molecule determines the chain length. It has been reported that plant stress resistance is closely related to chain length (23). In this study, 278 lipids with lipid chain lengths ranging from 10 to 75 were divided into 10 groups based on carbon chain length. With average contents of 1969.36, 1798.40 and 565.57 nmol/g, the top three chain length classes are 50–54, 35–39, and 30–34. When we compared the three types of oils, we discovered that the greatest variation (17.55%) was found for chain length ranged from 15 to 19. The oil extracted from the infested oilseeds (Type C) had the highest contents in the majority of the chain-length groups, especially for chains longer than 45 (Figure 3A). Type C had a chain length range of 20–24 of 17.33 nmol/g, which was 1.17 and 1.12 times higher than oil extracted from non-infested oilseeds of non-infested fruit (Type A) and non-infested oilseeds of infested fruit (Type B), respectively. Moreover, Type C was significantly (*p* < 0.05) higher in the chain length ranges of 45–49 and 55–59 at 23.28 nmol/g and 150.92 nmol/g, respectively, than oil extracted from non-infested oilseeds. The average content of oil extracted from non-infested oilseeds at chain lengths of 15–19 was 287.09 nmol/g, which was significantly (*p* < 0.05) higher than the oil extracted from the infested oilseeds. There was, however, no significant (*p* > 0.05) difference between the two oils extracted from non-infested oilseeds for all chain length groups.

### Effect of insect damage on lipid chain unsaturation of camellia oil

Chain unsaturation is defined as the sum of the number of double bonds in a lipid molecule’s fatty acid chains. The degree of unsaturation of lipids has been shown to be biologically important in stress response (24). As a result, the current study took into account the content of lipid compounds with the same number of unsaturated bonds. The differences in lipid molecule content among the three types of oilseeds were further compared at each degree of unsaturation (Figure 3B). Except for unsaturation 0 and 4, the highest contents of all degrees of unsaturation were found in oil extracted from the infested oilseeds (Type C). At chain unsaturation degrees of 5, 6, 7, 8, and 10, Type C was significantly (*p* < 0.05) higher than both types of oils extracted from the non-infested oilseeds (Type A and Type B). In addition, at chain unsaturation degree 10, Type C is 2.1 times higher than oil extracted from non-infested oilseeds of non-infested fruits (Type A). However, oil extracted from the non-infested oilseeds of the infested fruits (Type B) contained the most saturated lipids at 570.88 nmol/g, compared to 559.34 nmol/g and 511.28 nmol/g for Type A and Type C, respectively. Similarly, at various degrees of unsaturation, the two types of oils extracted from non-infested oilseeds did not show significant (*p* > 0.05) differences.

### Multivariate statistical analysis

**FIGURE 4:**
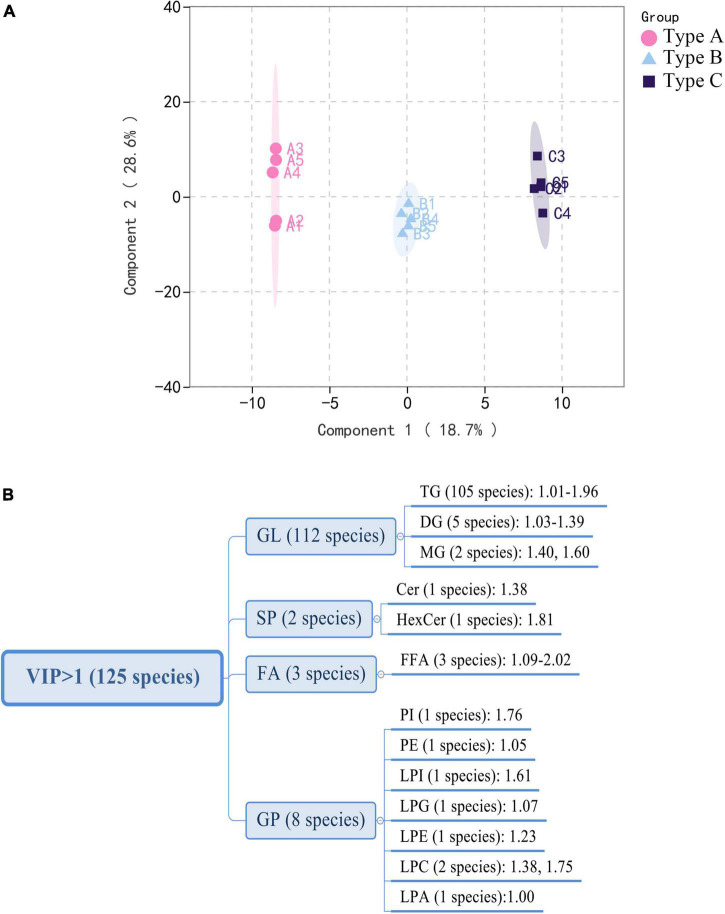
Multivariate analysis of lipidomics distinguishes three types of oils. **(A)** PLS-DA scores plot. **(B)** VIP scores of individual lipids in PLS-DA. GL, glycerolipids; GP, glycerophospholipids; FA, fatty acyls; SP, sphingolipids.

Partial Least-Squares Discriminant Analysis (PLS-DA) was used in the current study to distinguish the three types of camellia oils based on lipids found in these samples. PLS-DA is a supervised pattern recognition method used in multivariate statistical analysis. The goal of PLS-DA is to maximize group differentiation, thereby facilitating the search for differential metabolites (10). Besides, the PLS models also provided theoption of obtaining a quantitative measure of each variable’s discriminating power via variable importance for the projection. According to the score plot of individual lipids (Figure 4A), three types of oils were clearly classified based on the first two principal components. Individual lipid VIP scores that contribute to oils differentiation were also estimated. As shown in Figure 4B, a total of 125 individual lipids variables were considered to have a significant contribution (VIP > 1). Among these 125 lipids, there were 105 TGs, 5 DGs, 3 free fatty acids (FFAs), and several other individual lipids [two MGs, two LPCs, one PI, one PE, one LPI, one lysophosphatidyl glycerol (LPG), one LPE, one LPA, one hexosaccharide ceramide (HexCer), one Cer]. Individual lipids could also be used to distinguish three types of oils.

## Discussion

**FIGURE 5:**
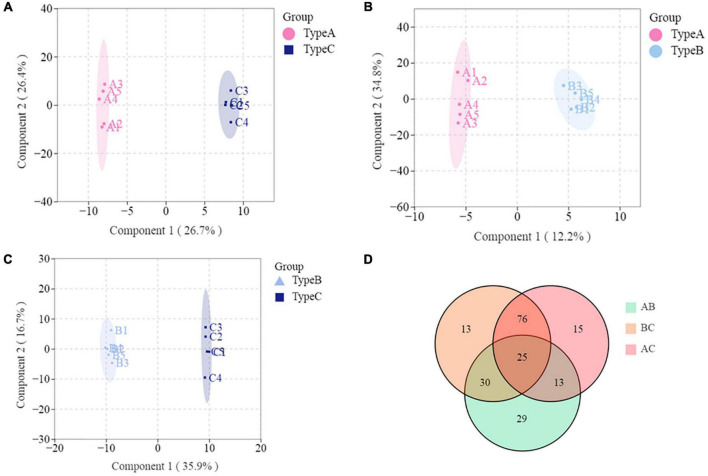
Multivariate analysis of lipidomics distinguishes any two types of oils. **(A)** PLS-DA scores plot based on Type A and Type C. **(B)** PLS-DA scores plot based on Type A and Type B. **(C)** PLS-DA scores plot based on Type B and Type C. **(D)** Venn diagram.

Lipids play a major role in life activities and have many important physiological functions. Some research has found that lipid chain length and unsaturation are closely related to abiotic stresses in plants (14, 23). To the best of our knowledge, no study has been published on the effects of lipid compositions other than fatty acids in response to biotic stress have been published (25–27). The current study found that oil extracted from infested oilseeds (Type C) contained significantly more long-chain lipids and unsaturated lipids than the two types of oils extracted from non-infested oilseeds (Type A and B). Especially, there is a highly significant (*p* < 0.01) increase in TG classes with chain-length > 45 and unsaturation ≥ 2 such as TG(16:0/23:0/18:2), TG(16:0/20:2/20:2) and TG(16:0/18:0/20:6). It was comparable to previous findings in Jatropha under cold stress (24) and Arabidopsis under heat stress (28). The findings might suggest that lipid unsaturation and chain length are also related to plant biotic stress, and that changes in the degree of unsaturation and length of lipids may be a common physiological basis for camellia fruit insect damage tolerance. The results of PLS-DA also demonstrated that Type C can be distinguished from Type A and B (Figures 5A,B). The number of individuals for those lipids that have effective discrimination (VIP > 1) of oil combinations was depicted in the Venn diagram (Figure 5D). It is worth noting that 101 of the lipids (VIP > 1) that significantly contributed to the discrimination of Type C and A, as well as Type C and B, were identical. Among these lipids, FFA (24:6) not only has the highest variation (76.15%), but also contributed most to both determinations, with average VIP value of 1.77. In comparison, the GL class that contributed the most to distinguishing lipid differences in peanut oil before and after roasting (VIP > 4) (14). This may be linked to the mechanism of lipid changes in response to various stresses. Moreover, we discovered an intriguing phenomenon: both Type B (from infested fruits) and Type A (from perfectly non-infested fruits) are extracted from non-infested oilseeds, they can be distinguished from each other (Figure 5C). The composition of LPC (16:3) was the main difference between them. This finding implies that insect damage may still have an effect on the lipid composition of uninfested seeds in the same fruit, possibly due to allelopathy effects (29).

**FIGURE 6:**
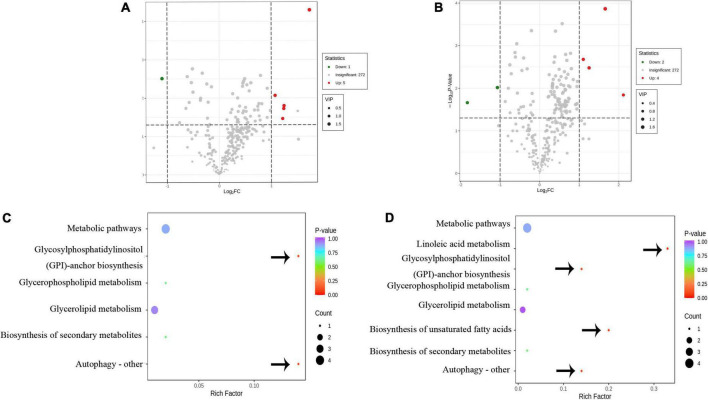
The differential metabolic analysis in response to insect damage. **(A)** Volcano diagram of the differential metabolites of Type A and Type C. **(B)** Volcano diagram of the differential metabolites of Type B and Type C. **(C)** Metabolic pathway diagram for differences in Type A and Type C. **(D)** Metabolic pathway diagram for differences in Type B and Type C. **(A,B)** Red and green dots represent up-regulated and down-regulated significant (fold change ≥ 2 or fold change ≤ 0.5, *p*-value < 0.05, and VIP ≥ 1) difference metabolites, respectively. **(C,D)** The bubble size represents the number of different metabolites involved in this pathway, and the bubble color represents the *p*-value of this metabolic pathway. The important metabolic pathways are pointed out by arrows.

To identify the biomarker for various abiotic stresses, many studies have established more stringent filtering conditions for screening differential metabolites, such as fold change ≥ 2 or fold change ≤ 0.5, *p*-value < 0.05, and VIP ≥ 1 (30, 31). Based on these biomarkers, possible metabolic pathways for edible oils when exposed to abiotic stresses would be clarified (15, 17). In this study, the amount of differential metabolites (lipids) between Type A and Type B under more stringent conditions was 0 to screen, indicating no significant differences in metabolite profiles between the two types of oils. In addition, a total of 10 differential metabolites were identified in the other two comparison groups, including 6 metabolites in each of the Type A vs. Type C and Type B vs. Type C groups (Figures 6A,B). Half of the differential metabolites were TG, which is similar to what was previously observed in the storage oxidation process of hazelnut oil (15). Notably, one FA [FFA(24:6)], a differential metabolite in our study that separated the two groups (Type A vs. Type C and Type B vs. Type C), is a class that has never been reported in the literature on abiotic stresses (15, 17). This could be due to the more noticeable impact of biotic stress on fatty acid changes. In addition, the effect of insect damage on the lipid metabolism of camellia oil was explored. For each of the two groups of significantly different lipids, we ran a KEGG enrichment analysis (Figures 6C,D). The metabolic pathways “Glycosylphosphatidylinositol anchor biosynthesis” and “Autophagy—other” had a *p*-value < 0.05, indicating that the Type A and Type C oils were significantly different in terms of the relative content of GP. In addition to these two metabolic pathways, the metabolic pathways “Linoleic acid metabolism” and “Biosynthesis of unsaturated fatty acids” were found to be significantly (*p* < 0.05) enriched only when Type B vs. Type C was compared. Overall, it is hypothesized that the four pathways mentioned above are more closely related to insect damage response.

## Conclusion

In this study, the changes in lipid profile of camellia oil obtained from infested and non-infested oil fruits were evaluated based on lipidomic approach. A total of 278 individual lipids from 6 lipid categories (FA, GL, GP, SP, ST, and PR) and 22 subclasses were identified in camellia oil. FFA(24:6) made the best commitment to the partition of oil extracted from infested oilseeds and two oils extracted from non-infested oilseeds. There was an exceptionally huge distinction in LPC (16:3) among oil extracted from non-infested oilseeds of non-infested fruits and oil extracted from non-infested oilseeds of infested fruits. In addition, ten significantly different lipids were then identified between infested and non-infested camellia oils by using bioinformatics analysis. Camellia oil’s four most important metabolic pathways that respond to insect damage were identified. The results of this study would help to understand the effect of biotic stress on changes in the lipid profile of camellia oil, as well as be useful for improving the nutritional quality of camellia oil.

## Data availability statement

The original contributions presented in this study are included in the article/Supplementary material, further inquiries can be directed to the corresponding author/s.

## Author contributions

QL: writing—original draft, conceptualization, visualization, investigation, and data curation. WZ: writing and investigation. DS: investigation and methodology. ZL: investigation and validation. JS: investigation. YL: writing—review and editing, conceptualization, software, and visualization. All authors contributed to the article and approved the submitted version.

## Footnotes

1
https://sciex.com.cn/

2
https://sciex.com/

3
http://www.metware.cn/
