# Supplementary material for: Comprehensive lipidomics analysis reveals the changes in lipid profile of camellia oil affected by insect damage
Source: Front Nutr. 2022 Sep 2;9:993334. doi: 10.3389/fnut.2022.993334 (PMC9478382; doi:10.3389/fnut.2022.993334)
Supplement: Supplementary file 1 [file Table_1.DOCX]

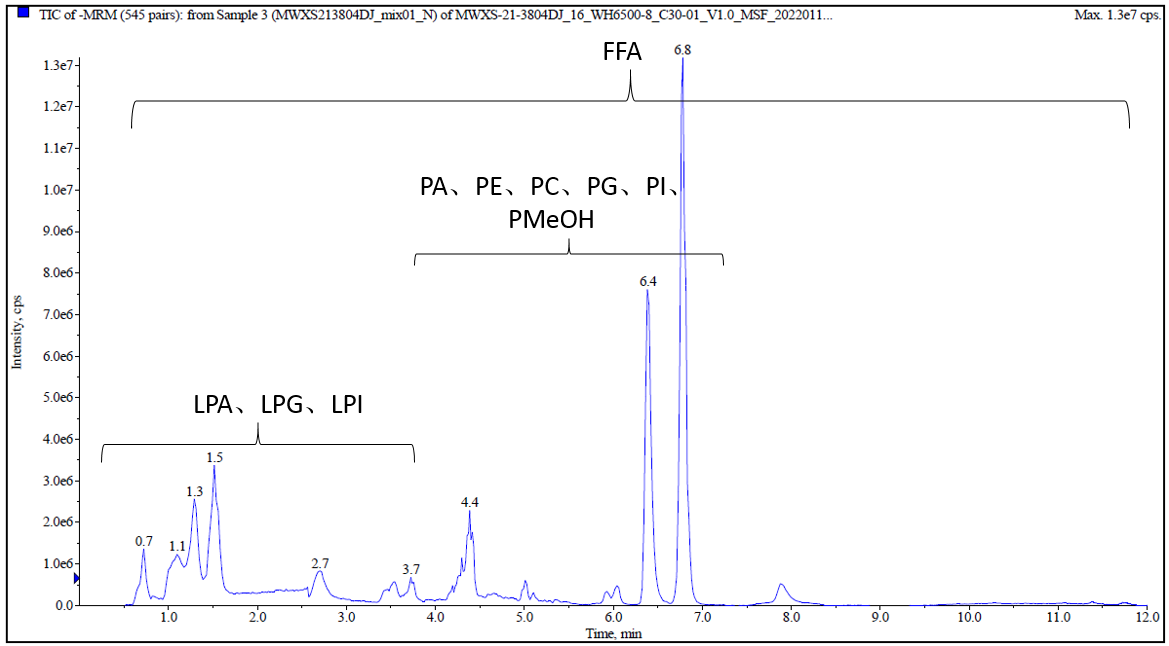
(a)


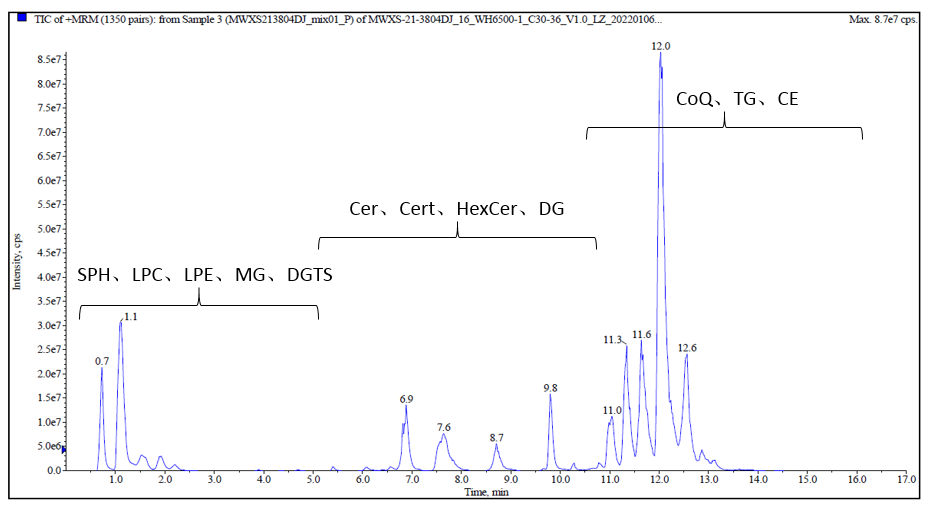
(b)

**Fig. S1. Total ion chromatogram of sample with information about elution order of different lipid classes.** (a) negative ion mode. (b) positive ion mode.

Note: FFA: free fatty acid; DG: Diacylglycerol; DGTs: Diacylglycerol trimethylhigh serine; TG: Triacylglycerol; MG: Monoacylglyceride; LPA: lysophosphatidic acids; LPC: lysophosphatidylcholine; LPE: Lysophosphatidylethanolamine; LPG: Lysophosphatidyl glycerol; LPI: Lysophosphatidylinositol; PA: Phosphatidic acid; PC: phosphatidylcholine; PE: phosphatidyl ethanolamine; PG: phosphatidylglycerol; PI: phosphatidylinositol; PMeOH: Phosphatidyl methanol; CoQ: Coenzyme Q10; Cer: ceramide; Cert: Ceramide transfer protein; Hexcer: Hexosaccharide ceramide; SPH: Sphingosine; CE: cholesteryl ester


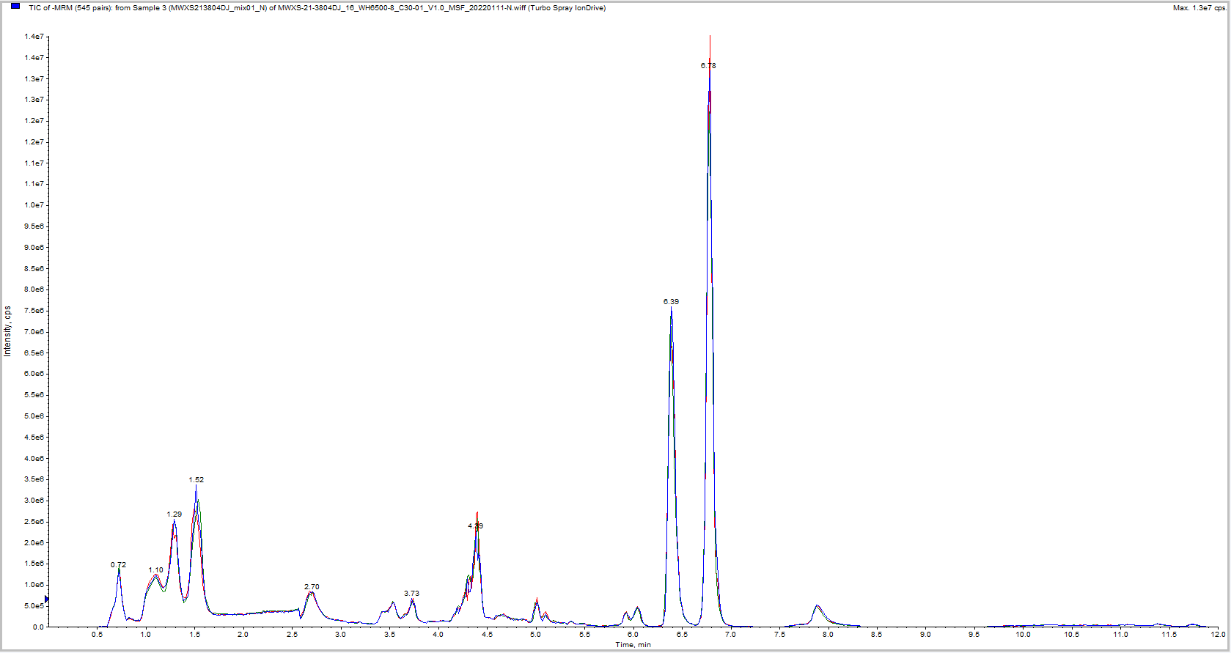
(a)


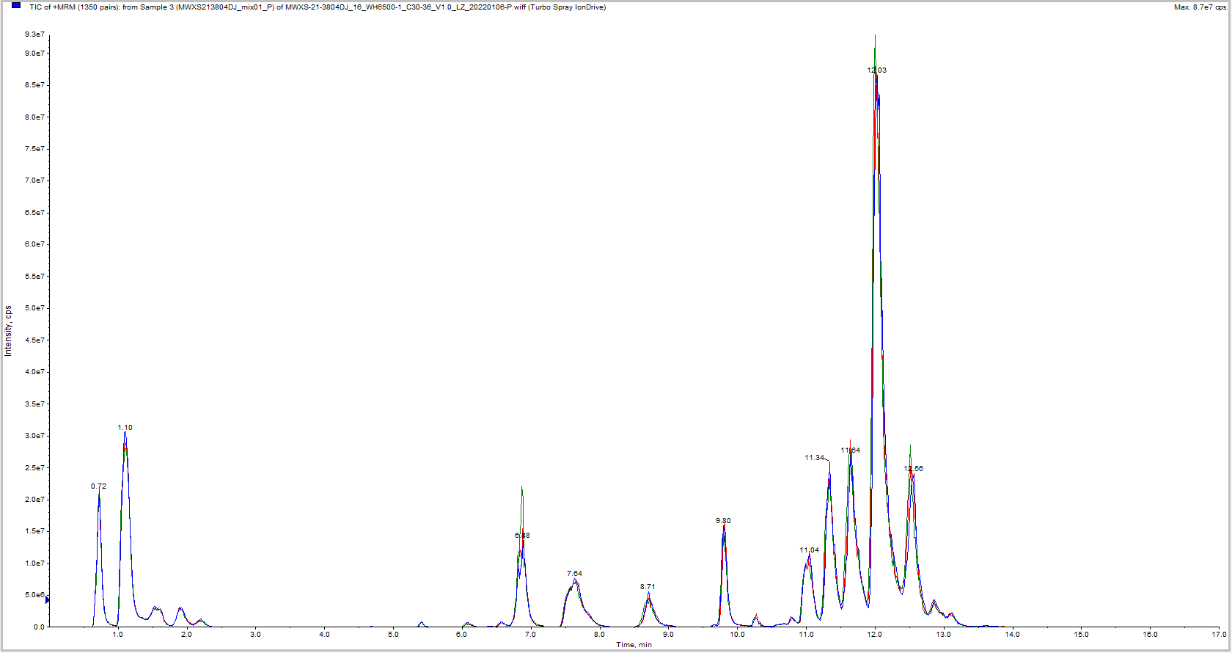
(b)

**Fig. S2. Overlay of the TIC**. (a) TIC overlap in negative ion mode. (b) TIC overlap in positive ion mode.

**Table S1 Information on the ion-pair, molecular weight, ionization model and formula of 278 lipids.**

| Number | Compounds | Class I | Class II | Q1 (Da) | Q3 | Molecular Weight | Ionization model | Formula |
| --- | --- | --- | --- | --- | --- | --- | --- | --- |
| 1 | CE(19:0) | ST | CE | 684.7 | 369.4 | 666.63 | [M+NH4]+ | C46H82O2 |
| 2 | Cer(d18:0/22:1(2OH)) | SP | Cer | 638.6 | 266.2 | 637.60 | [M+H]+ | C40H79NO4 |
| 3 | Cer(d18:1/24:0) | SP | Cer | 650.6 | 264.2 | 649.64 | [M+H]+ | C42H83NO3 |
| 4 | Cer(d18:1/16:1) | SP | Cer | 536.5 | 264.2 | 535.50 | [M+H]+ | C34H65NO3 |
| 5 | Cer(t18:0/18:0(2OH)) | SP | Cert | 600.6 | 264.2 | 599.55 | [M+H]+ | C36H73NO5 |
| 6 | Coenzyme Q10 | PR | CoQ | 863.7 | 197.1 | 862.68 | [M+H]+ | C59H90O4 |
| 7 | DG(18:2_20:1) | GL | DG | 664.6 | 337.3 | 646.55 | [M+NH4]+ | C41H74O5 |
| 8 | DG(16:0_20:0) | GL | DG | 642.6 | 313.3 | 624.57 | [M+NH4]+ | C39H76O5 |
| 9 | DG(18:0_18:0) | GL | DG | 642.6 | 341.3 | 624.57 | [M+NH4]+ | C39H76O5 |
| 10 | DG(14:0_20:0) | GL | DG | 614.6 | 285.2 | 596.54 | [M+NH4]+ | C37H72O5 |
| 11 | DG(16:0_16:0) | GL | DG | 586.5 | 313.3 | 568.51 | [M+NH4]+ | C35H68O5 |
| 12 | DG(14:0_18:0) | GL | DG | 586.5 | 285.2 | 568.51 | [M+NH4]+ | C35H68O5 |
| 13 | DG(14:0_22:0) | GL | DG | 642.6 | 285.2 | 624.57 | [M+NH4]+ | C39H76O5 |
| 14 | DG(8:0_16:0) | GL | DG | 474.4 | 201.1 | 456.38 | [M+NH4]+ | C27H52O5 |
| 15 | DG(18:0_18:3) | GL | DG | 636.6 | 341.3 | 618.52 | [M+NH4]+ | C39H70O5 |
| 16 | DG(18:1_18:2) | GL | DG | 636.6 | 337.3 | 618.52 | [M+NH4]+ | C39H70O5 |
| 17 | DG(16:0_18:0) | GL | DG | 614.6 | 341.3 | 596.54 | [M+NH4]+ | C37H72O5 |
| 18 | DG(18:2_22:1) | GL | DG | 692.6 | 337.3 | 674.58 | [M+NH4]+ | C43H78O5 |
| 19 | DG(20:0_18:0) | GL | DG | 670.6 | 369.3 | 652.60 | [M+NH4]+ | C41H80O5 |
| 20 | DG(16:0_18:1) | GL | DG | 612.6 | 313.3 | 594.52 | [M+NH4]+ | C37H70O5 |
| 21 | DG(16:1_20:2) | GL | DG | 636.6 | 365.3 | 618.52 | [M+NH4]+ | C39H70O5 |
| 22 | DG(17:1_18:2) | GL | DG | 622.5 | 337.3 | 604.51 | [M+NH4]+ | C38H68O5 |
| 23 | DG(16:0_18:3) | GL | DG | 608.5 | 335.3 | 590.49 | [M+NH4]+ | C37H66O5 |
| 24 | DG(16:1_18:2) | GL | DG | 608.5 | 311.3 | 590.49 | [M+NH4]+ | C37H66O5 |
| 25 | DG(18:2_24:0) | GL | DG | 722.7 | 425.4 | 704.63 | [M+NH4]+ | C45H84O5 |
| 26 | DG(18:2_22:0) | GL | DG | 694.6 | 337.3 | 676.60 | [M+NH4]+ | C43H80O5 |
| 27 | DG(18:1_20:1) | GL | DG | 666.6 | 339.3 | 648.57 | [M+NH4]+ | C41H76O5 |
| 28 | DG(18:1_18:1) | GL | DG | 638.6 | 339.3 | 620.54 | [M+NH4]+ | C39H72O5 |
| 29 | DG(18:0_18:2) | GL | DG | 638.6 | 341.3 | 620.54 | [M+NH4]+ | C39H72O5 |
| 30 | DG(17:1_18:1) | GL | DG | 624.6 | 325.3 | 606.52 | [M+NH4]+ | C38H70O5 |
| 31 | DG(16:0_18:2) | GL | DG | 610.5 | 313.3 | 592.51 | [M+NH4]+ | C37H68O5 |
| 32 | DG(16:1_18:1) | GL | DG | 610.5 | 339.3 | 592.51 | [M+NH4]+ | C37H68O5 |
| 33 | DG(14:0_18:2) | GL | DG | 582.5 | 337.3 | 564.48 | [M+NH4]+ | C35H64O5 |
| 34 | DG(18:0_18:1) | GL | DG | 640.6 | 341.3 | 622.55 | [M+NH4]+ | C39H74O5 |
| 35 | DG(17:0_18:1) | GL | DG | 626.6 | 327.3 | 608.54 | [M+NH4]+ | C38H72O5 |
| 36 | DG(14:0_16:1) | GL | DG | 556.5 | 311.3 | 538.46 | [M+NH4]+ | C33H62O5 |
| 37 | DG(16:0_20:4) | GL | DG | 634.5 | 313.3 | 616.51 | [M+NH4]+ | C39H68O5 |
| 38 | DG(14:1_22:2) | GL | DG | 636.6 | 283.2 | 618.52 | [M+NH4]+ | C39H70O5 |
| 39 | DG(18:3_18:3) | GL | DG | 630.5 | 335.3 | 612.48 | [M+NH4]+ | C39H64O5 |
| 40 | DG(18:2_18:3) | GL | DG | 632.5 | 337.3 | 614.49 | [M+NH4]+ | C39H66O5 |
| 41 | DG(18:2_18:2) | GL | DG | 634.5 | 337.3 | 616.51 | [M+NH4]+ | C39H68O5 |
| 42 | DG(16:1_20:3) | GL | DG | 634.5 | 363.3 | 616.51 | [M+NH4]+ | C39H68O5 |
| 43 | DG(18:1_18:3) | GL | DG | 634.5 | 339.3 | 616.51 | [M+NH4]+ | C39H68O5 |
| 44 | DGTS(14:0_18:4) | GL | DGTS | 704.5 | 446.3 | 703.54 | [M+H]+ | C42H73NO7 |
| 45 | FFA(18:2) | FA | FFA | 279.2 | 279.2 | 280.24 | [M-H]- | C18H32O2 |
| 46 | FFA(22:1) | FA | FFA | 337.3 | 337.3 | 338.32 | [M-H]- | C22H42O2 |
| 47 | FFA(18:1) | FA | FFA | 281.2 | 281.2 | 282.26 | [M-H]- | C18H34O2 |
| 48 | FFA(16:1) | FA | FFA | 253.2 | 253.2 | 254.22 | [M-H]- | C16H30O2 |
| 49 | FFA(36:0) | FA | FFA | 535.5 | 535.5 | 368.37 | [M-H]- | C36H72O2 |
| 50 | FFA(10:0) | FA | FFA | 171.1 | 171.1 | 172.15 | [M-H]- | C10H20O2 |
| 51 | FFA(24:6) | FA | FFA | 355.3 | 355.3 | 356.27 | [M-H]- | C24H36O2 |
| 52 | FFA(17:0) | FA | FFA | 269.2 | 269.2 | 270.26 | [M-H]- | C17H34O2 |
| 53 | FFA(16:0) | FA | FFA | 255.2 | 255.2 | 256.24 | [M-H]- | C16H32O2 |
| 54 | FFA(15:0) | FA | FFA | 241.2 | 241.2 | 242.22 | [M-H]- | C15H30O2 |
| 55 | FFA(14:0) | FA | FFA | 227.2 | 227.2 | 228.21 | [M-H]- | C14H28O2 |
| 56 | FFA(35:0) | FA | FFA | 521.5 | 521.5 | 368.37 | [M-H]- | C35H70O2 |
| 57 | FFA(18:0) | FA | FFA | 283.3 | 283.3 | 284.27 | [M-H]- | C18H36O2 |
| 58 | HexCer(d18:1/18:0) | SP | HexCer | 728.6 | 264.2 | 727.60 | [M+H]+ | C42H81NO8 |
| 59 | HexCer(d16:1/18:0) | SP | HexCer | 700.6 | 236.3 | 699.56 | [M+H]+ | C40H77NO8 |
| 60 | LPA(20:5) | GP | LPA | 455.2 | 153.0 | 456.23 | [M-H]- | C23H37O7P |
| 61 | LPA(16:3) | GP | LPA | 403.2 | 153.0 | 404.20 | [M-H]- | C19H33O7P |
| 62 | LPA(16:0) | GP | LPA | 409.2 | 153.0 | 410.24 | [M-H]- | C19H39O7P |
| 63 | LPA(18:0) | GP | LPA | 437.3 | 153.0 | 438.27 | [M-H]- | C21H43O7P |
| 64 | LPA(20:4) | GP | LPA | 457.2 | 153.0 | 458.24 | [M-H]- | C23H39O7P |
| 65 | LPC(16:0) | GP | LPC | 496.3 | 184.1 | 495.33 | [M+H]+ | C24H50NO7P |
| 66 | LPC(17:0) | GP | LPC | 510.4 | 184.1 | 509.35 | [M+H]+ | C25H52NO7P |
| 67 | LPC(16:3) | GP | LPC | 494.3 | 184.1 | 489.32 | [M+H]+ | C24H44NO7P |
| 68 | LPE(17:0) | GP | LPE | 468.3 | 327.3 | 467.30 | [M+H]+ | C22H46NO7P |
| 69 | LPE(18:1) | GP | LPE | 480.3 | 339.3 | 479.30 | [M+H]+ | C23H46NO7P |
| 70 | LPG(18:1) | GP | LPG | 509.3 | 281.2 | 510.30 | [M-H]- | C24H47O9P |
| 71 | LPG(16:0) | GP | LPG | 483.3 | 255.2 | 484.28 | [M-H]- | C22H45O9P |
| 72 | LPI(18:0) | GP | LPI | 599.3 | 283.3 | 600.33 | [M-H]- | C27H53O12P |
| 73 | LPI(18:1) | GP | LPI | 597.3 | 281.2 | 598.31 | [M-H]- | C27H51O12P |
| 74 | LPI(18:2) | GP | LPI | 595.3 | 279.2 | 596.30 | [M-H]- | C27H49O12P |
| 75 | LPI(22:6) | GP | LPI | 643.3 | 327.2 | 644.30 | [M-H]- | C31H49O12P |
| 76 | MG(16:0) | GL | MG | 348.3 | 239.3 | 330.28 | [M+NH4]+ | C19H38O4 |
| 77 | MG(18:0) | GL | MG | 376.4 | 267.3 | 358.31 | [M+NH4]+ | C21H42O4 |
| 78 | PA(18:1_22:6) | GP | PA | 745.5 | 281.2 | 746.49 | [M-H]- | C43H71O8P |
| 79 | PC(18:0_14:0) | GP | PC | 778.6 | 283.3 | 733.56 | [M+COOH]- | C40H80NO8P |
| 80 | PC(18:1_18:1) | GP | PC | 830.6 | 281.2 | 785.59 | [M+COOH]- | C44H84NO8P |
| 81 | PE(16:0_18:1) | GP | PE | 716.5 | 281.2 | 717.53 | [M-H]- | C39H76NO8P |
| 82 | PE(18:2_18:2) | GP | PE | 738.5 | 279.2 | 739.52 | [M-H]- | C41H74NO8P |
| 83 | PE(18:2_24:0) | GP | PE | 826.6 | 279.2 | 827.64 | [M-H]- | C47H90NO8P |
| 84 | PE(18:1_18:2) | GP | PE | 740.5 | 279.2 | 741.53 | [M-H]- | C41H76NO8P |
| 85 | PE(18:2_16:0) | GP | PE | 714.5 | 279.2 | 715.52 | [M-H]- | C39H74NO8P |
| 86 | PE(18:1_18:1) | GP | PE | 742.5 | 281.2 | 743.55 | [M-H]- | C41H78NO8P |
| 87 | PE(18:0_18:2) | GP | PE | 742.5 | 279.2 | 743.55 | [M-H]- | C41H78NO8P |
| 88 | PG(18:0_18:1) | GP | PG | 775.6 | 281.2 | 776.56 | [M-H]- | C42H81O10P |
| 89 | PG(16:0_18:1) | GP | PG | 747.5 | 255.2 | 748.53 | [M-H]- | C40H77O10P |
| 90 | PG(16:0_17:0) | GP | PG | 735.5 | 255.2 | 736.53 | [M-H]- | C39H77O10P |
| 91 | PI(18:1_18:1) | GP | PI | 861.6 | 281.2 | 862.56 | [M-H]- | C45H83O13P |
| 92 | PI(17:1_18:1) | GP | PI | 847.5 | 281.2 | 848.54 | [M-H]- | C44H81O13P |
| 93 | PI(16:1_18:1) | GP | PI | 833.5 | 281.2 | 834.53 | [M-H]- | C43H79O13P |
| 94 | PMeOH(16:0_22:5) | GP | PMeOH | 735.5 | 255.2 | 736.50 | [M-H]- | C42H73O8P |
| 95 | PMeOH(14:0_16:0) | GP | PMeOH | 633.4 | 227.2 | 634.46 | [M-H]- | C34H67O8P |
| 96 | SPH(d16:1) | SP | SPH | 272.3 | 254.3 | 271.25 | [M+H]+ | C16H33NO2 |
| 97 | PhytoSph(d16:2) | SP | SPH | 286.2 | 258.2 | 285.23 | [M+H]+ | C16H31NO3 |
| 98 | TG(18:1_18:2_20:0) | GL | TG | 930.8 | 633.6 | 912.81 | [M+NH4]+ | C59H108O6 |
| 99 | TG(16:0_18:2_20:2) | GL | TG | 900.8 | 575.5 | 882.77 | [M+NH4]+ | C57H102O6 |
| 100 | TG(18:0_18:1_18:3) | GL | TG | 900.8 | 605.6 | 882.77 | [M+NH4]+ | C57H102O6 |
| 101 | TG(18:1_19:1_18:2) | GL | TG | 914.8 | 601.5 | 896.78 | [M+NH4]+ | C58H104O6 |
| 102 | TG(16:0_20:2_20:2) | GL | TG | 928.8 | 603.5 | 910.80 | [M+NH4]+ | C59H106O6 |
| 103 | TG(18:1_18:3_20:0) | GL | TG | 928.8 | 599.5 | 910.80 | [M+NH4]+ | C59H106O6 |
| 104 | TG(18:0_18:3_20:1) | GL | TG | 928.8 | 601.5 | 910.80 | [M+NH4]+ | C59H106O6 |
| 105 | TG(18:1_18:1_20:2) | GL | TG | 928.8 | 629.6 | 910.80 | [M+NH4]+ | C59H106O6 |
| 106 | TG(20:0_18:2_18:2) | GL | TG | 928.8 | 631.6 | 910.80 | [M+NH4]+ | C59H106O6 |
| 107 | TG(18:1_22:1_18:2) | GL | TG | 956.9 | 601.5 | 938.83 | [M+NH4]+ | C61H110O6 |
| 108 | TG(18:1_18:3_22:0) | GL | TG | 956.9 | 599.5 | 938.83 | [M+NH4]+ | C61H110O6 |
| 109 | TG(20:1_20:1_18:2) | GL | TG | 956.9 | 629.5 | 938.83 | [M+NH4]+ | C61H110O6 |
| 110 | TG(23:0_18:2_18:2) | GL | TG | 970.9 | 599.5 | 952.85 | [M+NH4]+ | C62H112O6 |
| 111 | TG(18:1_24:0_18:3) | GL | TG | 984.9 | 599.5 | 966.86 | [M+NH4]+ | C63H114O6 |
| 112 | TG(18:2_24:0_18:2) | GL | TG | 984.9 | 687.6 | 966.86 | [M+NH4]+ | C63H114O6 |
| 113 | TG(25:0_18:2_18:2) | GL | TG | 998.9 | 701.6 | 980.88 | [M+NH4]+ | C64H116O6 |
| 114 | TG(20:1_24:1_18:2) | GL | TG | 1012.9 | 715.7 | 994.89 | [M+NH4]+ | C65H118O6 |
| 115 | TG(14:0_18:2_18:3) | GL | TG | 842.7 | 545.5 | 824.69 | [M+NH4]+ | C53H92O6 |
| 116 | TG(18:1_18:1_18:2) | GL | TG | 900.8 | 601.5 | 882.77 | [M+NH4]+ | C57H102O6 |
| 117 | TG(17:0_18:2_18:2) | GL | TG | 886.8 | 589.5 | 868.75 | [M+NH4]+ | C56H100O6 |
| 118 | TG(17:1_18:1_18:2) | GL | TG | 886.8 | 601.5 | 868.75 | [M+NH4]+ | C56H100O6 |
| 119 | TG(16:0_18:1_18:3) | GL | TG | 872.8 | 577.5 | 854.74 | [M+NH4]+ | C55H98O6 |
| 120 | TG(16:0_18:2_20:1) | GL | TG | 902.8 | 575.5 | 884.78 | [M+NH4]+ | C57H104O6 |
| 121 | TG(18:1_18:1_20:1) | GL | TG | 930.8 | 603.5 | 912.81 | [M+NH4]+ | C59H108O6 |
| 122 | TG(20:1_18:2_18:0) | GL | TG | 930.8 | 629.6 | 912.81 | [M+NH4]+ | C59H108O6 |
| 123 | TG(16:0_18:2_22:1) | GL | TG | 930.8 | 657.6 | 912.81 | [M+NH4]+ | C59H108O6 |
| 124 | TG(18:0_18:3_20:0) | GL | TG | 930.8 | 601.5 | 912.81 | [M+NH4]+ | C59H108O6 |
| 125 | TG(18:1_18:2_22:0) | GL | TG | 958.9 | 601.5 | 940.85 | [M+NH4]+ | C61H112O6 |
| 126 | TG(18:1_18:1_22:1) | GL | TG | 958.9 | 659.6 | 940.85 | [M+NH4]+ | C61H112O6 |
| 127 | TG(23:0_18:1_18:2) | GL | TG | 972.9 | 601.5 | 954.86 | [M+NH4]+ | C62H114O6 |
| 128 | TG(18:1_18:1_19:1) | GL | TG | 916.8 | 603.5 | 898.80 | [M+NH4]+ | C58H106O6 |
| 129 | TG(24:0_18:1_18:2) | GL | TG | 986.9 | 601.5 | 968.88 | [M+NH4]+ | C63H116O6 |
| 130 | TG(14:0_18:2_18:2) | GL | TG | 844.7 | 599.5 | 826.71 | [M+NH4]+ | C53H94O6 |
| 131 | TG(16:1_16:1_18:2) | GL | TG | 844.7 | 573.5 | 826.71 | [M+NH4]+ | C53H94O6 |
| 132 | TG(14:1_18:2_18:2) | GL | TG | 842.7 | 599.5 | 824.69 | [M+NH4]+ | C53H92O6 |
| 133 | TG(14:0_18:1_18:3) | GL | TG | 844.7 | 545.5 | 826.71 | [M+NH4]+ | C53H94O6 |
| 134 | TG(16:1_16:2_18:1) | GL | TG | 844.7 | 575.5 | 826.71 | [M+NH4]+ | C53H94O6 |
| 135 | TG(16:0_16:2_18:2) | GL | TG | 844.7 | 547.5 | 826.71 | [M+NH4]+ | C53H94O6 |
| 136 | TG(15:0_18:2_18:2) | GL | TG | 858.8 | 561.5 | 840.72 | [M+NH4]+ | C54H96O6 |
| 137 | TG(16:0_18:2_18:2) | GL | TG | 872.8 | 599.5 | 854.74 | [M+NH4]+ | C55H98O6 |
| 138 | TG(18:2_20:1_22:0) | GL | TG | 986.9 | 689.6 | 968.88 | [M+NH4]+ | C63H116O6 |
| 139 | TG(16:0_16:1_18:3) | GL | TG | 844.7 | 571.5 | 826.71 | [M+NH4]+ | C53H94O6 |
| 140 | TG(14:0_16:0_22:5) | GL | TG | 870.8 | 523.5 | 852.72 | [M+NH4]+ | C55H96O6 |
| 141 | TG(15:0_18:2_18:3) | GL | TG | 856.7 | 559.5 | 838.71 | [M+NH4]+ | C54H94O6 |
| 142 | TG(16:0_18:3_18:3) | GL | TG | 868.7 | 573.5 | 850.71 | [M+NH4]+ | C55H94O6 |
| 143 | TG(16:2_18:1_18:3) | GL | TG | 868.7 | 599.5 | 850.71 | [M+NH4]+ | C55H94O6 |
| 144 | TG(17:0_18:3_18:3) | GL | TG | 882.8 | 587.5 | 864.72 | [M+NH4]+ | C56H96O6 |
| 145 | TG(18:1_18:2_18:3) | GL | TG | 896.8 | 601.5 | 878.74 | [M+NH4]+ | C57H98O6 |
| 146 | TG(18:2_18:2_18:2) | GL | TG | 896.8 | 599.5 | 878.74 | [M+NH4]+ | C57H98O6 |
| 147 | TG(16:0_18:0_20:6) | GL | TG | 896.8 | 579.5 | 878.74 | [M+NH4]+ | C57H98O6 |
| 148 | TG(18:1_18:1_18:4) | GL | TG | 896.8 | 597.5 | 878.74 | [M+NH4]+ | C57H98O6 |
| 149 | TG(18:2_18:2_20:2) | GL | TG | 924.8 | 627.5 | 906.77 | [M+NH4]+ | C59H102O6 |
| 150 | TG(18:2_18:3_20:1) | GL | TG | 924.8 | 597.5 | 906.77 | [M+NH4]+ | C59H102O6 |
| 151 | TG(24:1_18:2_18:3) | GL | TG | 980.9 | 597.5 | 962.83 | [M+NH4]+ | C63H110O6 |
| 152 | TG(18:2_18:2_18:3) | GL | TG | 894.8 | 599.5 | 876.72 | [M+NH4]+ | C57H96O6 |
| 153 | TG(18:1_18:2_18:4) | GL | TG | 894.8 | 595.5 | 876.72 | [M+NH4]+ | C57H96O6 |
| 154 | TG(16:4_18:1_18:3) | GL | TG | 864.7 | 565.4 | 846.67 | [M+NH4]+ | C55H90O6 |
| 155 | TG(18:2_18:3_18:3) | GL | TG | 892.7 | 597.5 | 874.71 | [M+NH4]+ | C57H94O6 |
| 156 | TG(18:1_18:2_20:5) | GL | TG | 920.8 | 621.5 | 902.74 | [M+NH4]+ | C59H98O6 |
| 157 | TG(16:0_20:3_20:5) | GL | TG | 920.8 | 601.5 | 902.74 | [M+NH4]+ | C59H98O6 |
| 158 | TG(18:3_18:3_18:3) | GL | TG | 890.7 | 595.5 | 872.69 | [M+NH4]+ | C57H92O6 |
| 159 | TG(18:2_18:4_18:4) | GL | TG | 888.7 | 591.4 | 870.67 | [M+NH4]+ | C57H90O6 |
| 160 | TG(16:3_18:2_22:5) | GL | TG | 916.7 | 619.5 | 898.71 | [M+NH4]+ | C59H94O6 |
| 161 | TG(16:1_16:1_20:4) | GL | TG | 868.7 | 597.5 | 850.71 | [M+NH4]+ | C55H94O6 |
| 162 | TG(14:0_14:0_22:5) | GL | TG | 842.7 | 597.5 | 824.69 | [M+NH4]+ | C53H92O6 |
| 163 | TG(16:0_16:1_20:5) | GL | TG | 868.7 | 595.5 | 850.71 | [M+NH4]+ | C55H94O6 |
| 164 | TG(18:2_18:2_22:1) | GL | TG | 954.8 | 657.6 | 936.81 | [M+NH4]+ | C61H108O6 |
| 165 | TG(15:1_18:2_18:2) | GL | TG | 856.7 | 599.5 | 838.71 | [M+NH4]+ | C54H94O6 |
| 166 | TG(16:1_18:2_18:2) | GL | TG | 870.8 | 599.5 | 852.72 | [M+NH4]+ | C55H96O6 |
| 167 | TG(16:0_18:2_18:3) | GL | TG | 870.8 | 573.5 | 852.72 | [M+NH4]+ | C55H96O6 |
| 168 | TG(18:0_18:0_18:3) | GL | TG | 902.8 | 601.5 | 884.78 | [M+NH4]+ | C57H104O6 |
| 169 | TG(16:3_18:1_18:1) | GL | TG | 870.8 | 571.5 | 852.72 | [M+NH4]+ | C55H96O6 |
| 170 | TG(16:0_18:1_18:4) | GL | TG | 870.8 | 577.5 | 852.72 | [M+NH4]+ | C55H96O6 |
| 171 | TG(17:1_18:2_18:2) | GL | TG | 884.8 | 599.5 | 866.74 | [M+NH4]+ | C56H98O6 |
| 172 | TG(17:1_18:1_18:3) | GL | TG | 884.8 | 585.5 | 866.74 | [M+NH4]+ | C56H98O6 |
| 173 | TG(18:1_18:2_18:2) | GL | TG | 898.8 | 601.5 | 880.75 | [M+NH4]+ | C57H100O6 |
| 174 | TG(18:0_18:2_18:3) | GL | TG | 898.8 | 603.5 | 880.75 | [M+NH4]+ | C57H100O6 |
| 175 | TG(16:0_18:3_20:2) | GL | TG | 898.8 | 573.5 | 880.75 | [M+NH4]+ | C57H100O6 |
| 176 | TG(18:0_18:1_18:4) | GL | TG | 898.8 | 605.6 | 880.75 | [M+NH4]+ | C57H100O6 |
| 177 | TG(18:1_18:1_18:3) | GL | TG | 898.8 | 599.5 | 880.75 | [M+NH4]+ | C57H100O6 |
| 178 | TG(17:0_18:1_20:4) | GL | TG | 912.8 | 613.5 | 894.77 | [M+NH4]+ | C58H102O6 |
| 179 | TG(19:1_18:2_18:2) | GL | TG | 912.8 | 615.5 | 894.77 | [M+NH4]+ | C58H102O6 |
| 180 | TG(18:1_18:2_20:2) | GL | TG | 926.8 | 627.5 | 908.78 | [M+NH4]+ | C59H104O6 |
| 181 | TG(18:0_18:3_20:2) | GL | TG | 926.8 | 601.5 | 908.78 | [M+NH4]+ | C59H104O6 |
| 182 | TG(18:2_18:2_20:1) | GL | TG | 926.8 | 629.5 | 908.78 | [M+NH4]+ | C59H104O6 |
| 183 | TG(18:1_18:3_20:1) | GL | TG | 926.8 | 599.5 | 908.78 | [M+NH4]+ | C59H104O6 |
| 184 | TG(22:1_18:2_20:2) | GL | TG | 982.9 | 685.6 | 964.85 | [M+NH4]+ | C63H112O6 |
| 185 | TG(16:0_18:1_20:2) | GL | TG | 902.8 | 629.6 | 884.78 | [M+NH4]+ | C57H104O6 |
| 186 | TG(16:1_18:1_18:1) | GL | TG | 874.8 | 603.5 | 856.75 | [M+NH4]+ | C55H100O6 |
| 187 | TG(18:1_18:1_18:1) | GL | TG | 902.8 | 603.5 | 884.78 | [M+NH4]+ | C57H104O6 |
| 188 | TG(16:0_17:0_18:1) | GL | TG | 864.8 | 577.5 | 846.77 | [M+NH4]+ | C54H102O6 |
| 189 | TG(16:0_18:0_18:1) | GL | TG | 878.8 | 577.5 | 860.78 | [M+NH4]+ | C55H104O6 |
| 190 | TG(17:0_18:0_18:1) | GL | TG | 892.8 | 591.5 | 874.80 | [M+NH4]+ | C56H106O6 |
| 191 | TG(18:0_18:0_18:1) | GL | TG | 906.8 | 605.6 | 888.81 | [M+NH4]+ | C57H108O6 |
| 192 | TG(16:0_20:0_18:1) | GL | TG | 906.8 | 577.5 | 888.81 | [M+NH4]+ | C57H108O6 |
| 193 | TG(16:0_18:1_20:0) | GL | TG | 906.8 | 607.6 | 888.81 | [M+NH4]+ | C57H108O6 |
| 194 | TG(18:0_18:1_20:0) | GL | TG | 934.9 | 635.6 | 916.85 | [M+NH4]+ | C59H112O6 |
| 195 | TG(16:0_18:1_22:0) | GL | TG | 934.9 | 661.6 | 916.85 | [M+NH4]+ | C59H112O6 |
| 196 | TG(16:0_20:0_20:1) | GL | TG | 934.9 | 605.6 | 916.85 | [M+NH4]+ | C59H112O6 |
| 197 | TG(15:0_24:0_18:1) | GL | TG | 948.9 | 649.6 | 930.86 | [M+NH4]+ | C60H114O6 |
| 198 | TG(16:0_23:0_18:1) | GL | TG | 948.9 | 675.6 | 930.86 | [M+NH4]+ | C60H114O6 |
| 199 | TG(16:0_24:0_18:1) | GL | TG | 962.9 | 663.6 | 944.88 | [M+NH4]+ | C61H116O6 |
| 200 | TG(16:0_25:0_18:1) | GL | TG | 976.9 | 577.5 | 958.89 | [M+NH4]+ | C62H118O6 |
| 201 | TG(16:0_26:0_18:1) | GL | TG | 990.9 | 577.5 | 972.91 | [M+NH4]+ | C63H120O6 |
| 202 | TG(18:0_24:0_18:1) | GL | TG | 990.9 | 689.6 | 972.91 | [M+NH4]+ | C63H120O6 |
| 203 | TG(12:0_16:1_18:1) | GL | TG | 792.7 | 493.4 | 774.67 | [M+NH4]+ | C49H90O6 |
| 204 | TG(16:0_16:1_16:1) | GL | TG | 820.7 | 547.5 | 802.71 | [M+NH4]+ | C51H94O6 |
| 205 | TG(14:0_16:0_18:2) | GL | TG | 820.7 | 523.5 | 802.71 | [M+NH4]+ | C51H94O6 |
| 206 | TG(14:0_16:1_18:1) | GL | TG | 820.7 | 521.5 | 802.71 | [M+NH4]+ | C51H94O6 |
| 207 | TG(16:0_16:0_18:1) | GL | TG | 850.8 | 551.5 | 832.75 | [M+NH4]+ | C53H100O6 |
| 208 | TG(14:1_16:0_18:1) | GL | TG | 820.7 | 577.5 | 802.71 | [M+NH4]+ | C51H94O6 |
| 209 | TG(16:0_16:1_17:0) | GL | TG | 836.8 | 549.5 | 818.74 | [M+NH4]+ | C52H98O6 |
| 210 | TG(15:0_16:0_18:1) | GL | TG | 836.8 | 577.5 | 818.74 | [M+NH4]+ | C52H98O6 |
| 211 | TG(10:0_12:0_14:0) | GL | TG | 656.6 | 467.4 | 638.55 | [M+NH4]+ | C39H74O6 |
| 212 | TG(13:0_14:0_16:0) | GL | TG | 754.7 | 509.5 | 736.66 | [M+NH4]+ | C46H88O6 |
| 213 | TG(14:0_14:0_16:0) | GL | TG | 768.7 | 523.5 | 750.67 | [M+NH4]+ | C47H90O6 |
| 214 | TG(14:0_15:0_16:0) | GL | TG | 782.7 | 537.5 | 764.69 | [M+NH4]+ | C48H92O6 |
| 215 | TG(14:0_16:0_16:0) | GL | TG | 796.7 | 523.7 | 778.71 | [M+NH4]+ | C49H94O6 |
| 216 | TG(15:0_16:0_16:0) | GL | TG | 810.8 | 537.5 | 792.72 | [M+NH4]+ | C50H96O6 |
| 217 | TG(14:0_16:0_17:0) | GL | TG | 810.8 | 523.5 | 792.72 | [M+NH4]+ | C50H96O6 |
| 218 | TG(16:0_16:0_16:0) | GL | TG | 824.8 | 551.5 | 806.74 | [M+NH4]+ | C51H98O6 |
| 219 | TG(16:0_16:0_17:0) | GL | TG | 838.8 | 565.5 | 820.75 | [M+NH4]+ | C52H100O6 |
| 220 | TG(16:0_16:0_18:0) | GL | TG | 852.8 | 579.5 | 834.77 | [M+NH4]+ | C53H102O6 |
| 221 | TG(16:0_18:0_18:0) | GL | TG | 880.8 | 579.8 | 862.80 | [M+NH4]+ | C55H106O6 |
| 222 | TG(16:0_16:0_20:0) | GL | TG | 880.8 | 607.6 | 862.80 | [M+NH4]+ | C55H106O6 |
| 223 | TG(17:0_17:0_19:0) | GL | TG | 894.8 | 607.6 | 876.81 | [M+NH4]+ | C56H108O6 |
| 224 | TG(18:0_18:0_18:0) | GL | TG | 908.9 | 607.6 | 890.83 | [M+NH4]+ | C57H110O6 |
| 225 | TG(12:0_16:0_18:1) | GL | TG | 794.7 | 577.5 | 776.69 | [M+NH4]+ | C49H92O6 |
| 226 | TG(14:0_16:0_16:1) | GL | TG | 794.7 | 523.5 | 776.69 | [M+NH4]+ | C49H92O6 |
| 227 | TG(15:0_16:0_16:1) | GL | TG | 808.7 | 537.5 | 790.71 | [M+NH4]+ | C50H94O6 |
| 228 | TG(16:0_16:0_16:1) | GL | TG | 822.8 | 549.5 | 804.72 | [M+NH4]+ | C51H96O6 |
| 229 | TG(14:0_16:0_18:1) | GL | TG | 822.8 | 523.5 | 804.72 | [M+NH4]+ | C51H96O6 |
| 230 | TG(16:0_16:0_17:1) | GL | TG | 836.8 | 563.5 | 818.74 | [M+NH4]+ | C52H98O6 |
| 231 | TG(16:0_16:1_17:1) | GL | TG | 834.8 | 561.5 | 816.72 | [M+NH4]+ | C52H96O6 |
| 232 | TG(15:0_16:0_18:2) | GL | TG | 834.8 | 575.5 | 816.72 | [M+NH4]+ | C52H96O6 |
| 233 | TG(13:0_18:1_18:1) | GL | TG | 834.8 | 535.5 | 816.72 | [M+NH4]+ | C52H96O6 |
| 234 | TG(18:0_22:0_18:2) | GL | TG | 960.9 | 663.6 | 942.86 | [M+NH4]+ | C61H114O6 |
| 235 | TG(16:0_18:1_24:1) | GL | TG | 960.9 | 577.5 | 942.86 | [M+NH4]+ | C61H114O6 |
| 236 | TG(16:0_24:0_18:2) | GL | TG | 960.9 | 575.5 | 942.86 | [M+NH4]+ | C61H114O6 |
| 237 | TG(18:0_20:0_20:2) | GL | TG | 960.9 | 659.6 | 942.86 | [M+NH4]+ | C61H114O6 |
| 238 | TG(18:1_20:0_22:1) | GL | TG | 960.9 | 605.6 | 942.86 | [M+NH4]+ | C61H114O6 |
| 239 | TG(24:0_18:1_18:1) | GL | TG | 988.9 | 603.5 | 970.89 | [M+NH4]+ | C63H118O6 |
| 240 | TG(26:0_18:1_18:1) | GL | TG | 1017.0 | 717.7 | 998.92 | [M+NH4]+ | C65H122O6 |
| 241 | TG(14:0_16:1_18:2) | GL | TG | 818.7 | 547.5 | 800.69 | [M+NH4]+ | C51H92O6 |
| 242 | TG(16:0_15:1_18:2) | GL | TG | 832.7 | 559.5 | 814.71 | [M+NH4]+ | C52H94O6 |
| 243 | TG(16:1_16:1_18:1) | GL | TG | 846.8 | 575.5 | 828.72 | [M+NH4]+ | C53H96O6 |
| 244 | TG(16:0_16:1_18:2) | GL | TG | 846.8 | 549.5 | 828.72 | [M+NH4]+ | C53H96O6 |
| 245 | TG(16:0_16:0_18:3) | GL | TG | 846.8 | 551.5 | 828.72 | [M+NH4]+ | C53H96O6 |
| 246 | TG(14:0_18:1_18:2) | GL | TG | 846.8 | 601.5 | 828.72 | [M+NH4]+ | C53H96O6 |
| 247 | TG(15:0_18:1_18:2) | GL | TG | 860.8 | 601.5 | 842.74 | [M+NH4]+ | C54H98O6 |
| 248 | TG(16:0_17:1_18:2) | GL | TG | 860.8 | 575.5 | 842.74 | [M+NH4]+ | C54H98O6 |
| 249 | TG(16:0_18:1_18:2) | GL | TG | 874.8 | 577.5 | 856.75 | [M+NH4]+ | C55H100O6 |
| 250 | TG(14:0_18:1_20:2) | GL | TG | 874.8 | 549.5 | 856.75 | [M+NH4]+ | C55H100O6 |
| 251 | TG(16:0_18:0_18:3) | GL | TG | 874.8 | 573.5 | 856.75 | [M+NH4]+ | C55H100O6 |
| 252 | TG(17:0_18:1_18:2) | GL | TG | 888.8 | 591.5 | 870.77 | [M+NH4]+ | C56H102O6 |
| 253 | TG(22:0_18:1_18:1) | GL | TG | 960.9 | 603.5 | 942.86 | [M+NH4]+ | C61H114O6 |
| 254 | TG(16:0_23:0_18:2) | GL | TG | 946.9 | 649.6 | 928.85 | [M+NH4]+ | C60H112O6 |
| 255 | TG(15:0_18:1_24:1) | GL | TG | 946.9 | 647.6 | 928.85 | [M+NH4]+ | C60H112O6 |
| 256 | TG(18:1_18:1_20:0) | GL | TG | 932.9 | 633.6 | 914.83 | [M+NH4]+ | C59H110O6 |
| 257 | TG(16:0_16:1_18:1) | GL | TG | 848.8 | 549.5 | 830.74 | [M+NH4]+ | C53H98O6 |
| 258 | TG(14:0_16:1_20:1) | GL | TG | 848.8 | 577.5 | 830.74 | [M+NH4]+ | C53H98O6 |
| 259 | TG(16:0_16:0_18:2) | GL | TG | 848.8 | 575.5 | 830.74 | [M+NH4]+ | C53H98O6 |
| 260 | TG(16:0_17:1_18:1) | GL | TG | 862.8 | 563.5 | 844.75 | [M+NH4]+ | C54H100O6 |
| 261 | TG(16:0_17:0_18:2) | GL | TG | 862.8 | 565.5 | 844.75 | [M+NH4]+ | C54H100O6 |
| 262 | TG(16:0_18:1_18:1) | GL | TG | 876.8 | 577.5 | 858.77 | [M+NH4]+ | C55H102O6 |
| 263 | TG(16:0_18:0_18:2) | GL | TG | 876.8 | 575.5 | 858.77 | [M+NH4]+ | C55H102O6 |
| 264 | TG(16:0_16:0_20:2) | GL | TG | 876.8 | 603.5 | 858.77 | [M+NH4]+ | C55H102O6 |
| 265 | TG(17:0_18:1_18:1) | GL | TG | 890.8 | 603.5 | 872.78 | [M+NH4]+ | C56H104O6 |
| 266 | TG(18:0_18:1_18:2) | GL | TG | 902.8 | 605.6 | 884.78 | [M+NH4]+ | C57H104O6 |
| 267 | TG(18:0_18:1_18:1) | GL | TG | 904.8 | 605.6 | 886.80 | [M+NH4]+ | C57H106O6 |
| 268 | TG(16:1_18:0_20:1) | GL | TG | 904.8 | 577.5 | 886.80 | [M+NH4]+ | C57H106O6 |
| 269 | TG(16:0_18:2_20:0) | GL | TG | 904.8 | 575.5 | 886.80 | [M+NH4]+ | C57H106O6 |
| 270 | TG(18:0_18:0_18:2) | GL | TG | 904.8 | 603.5 | 886.80 | [M+NH4]+ | C57H106O6 |
| 271 | TG(15:0_18:1_22:1) | GL | TG | 918.8 | 619.6 | 900.81 | [M+NH4]+ | C58H108O6 |
| 272 | TG(18:0_18:1_19:1) | GL | TG | 918.8 | 605.6 | 900.81 | [M+NH4]+ | C58H108O6 |
| 273 | TG(18:0_18:1_20:1) | GL | TG | 932.9 | 605.6 | 914.83 | [M+NH4]+ | C59H110O6 |
| 274 | TG(18:0_18:2_20:0) | GL | TG | 932.9 | 603.5 | 914.83 | [M+NH4]+ | C59H110O6 |
| 275 | TG(16:0_18:1_22:1) | GL | TG | 932.9 | 577.5 | 914.83 | [M+NH4]+ | C59H110O6 |
| 276 | TG(16:0_18:2_22:0) | GL | TG | 932.9 | 635.6 | 914.83 | [M+NH4]+ | C59H110O6 |
| 277 | TG(16:0_18:1_20:1) | GL | TG | 904.8 | 631.6 | 886.80 | [M+NH4]+ | C57H106O6 |
| 278 | TG(16:1_16:1_17:0) | GL | TG | 834.8 | 563.5 | 816.72 | [M+NH4]+ | C52H96O6 |

**Table S2** **Information on internal standards**

| **Number** | **Glass** | **Corresponding internal labels** | **CAS** | **Production** | **Detection mode** |
| --- | --- | --- | --- | --- | --- |
| 1 | LPA | LPA(17:0) | 799268-66-9 | Avanti | negative ion mode |
| 2 | LPC | LPC(16:0)-d31 | 327178-91-6 | Avanti | Positive ion mode |
| 3 | LPE | LPE(14:0) | 123060-40-2 | Avanti | Positive ion mode |
| 4 | LPG | LPG(14:0) | 123060-40-2 | Avanti | negative ion mode |
| 5 | LPI | LPI(17:1) | 1246353-39-8 | Avanti | negative ion mode |
| 6 | LPS | LPS(17:1) | 1246298-15-6 | Avanti | negative ion mode |
| 7 | PA | PA(17:0/17:0) | 154804-54-3 | Avanti | negative ion mode |
| 8 | PC | PC(16:0(d31)/18:1) | 179093-76-6 | Avanti | negative ion mode |
| 9 | PE | PE(16:0(d31)/18:1) | 326495-44-7 | Avanti | negative ion mode |
| 10 | PG | PG(16:0(d31)/18:1) | 327178-87-0 | Avanti | negative ion mode |
| 11 | PI | PI(16:0(d31)/18:1) | 799812-61-6 | Avanti | negative ion mode |
| 12 | PS | PS(16:0(d31)/18:1) | 327178-96-1 | Avanti | negative ion mode |
| 13 | PMeOH | PMeOH(16:0/16:0) | 92609-89-7 | zzstandard | negative ion mode |
| 14 | MG | MG(17:0) | 5638-14-2 | Sigma-Aldrich | Positive ion mode |
| 15 | DG | DG(17:0/17:0) | 98896-81-2 | Cayman | Positive ion mode |
| 16 | TG | TG(17:0/17:0/17:0) | 2438-40-6 | sigma-Aldrich | Positive ion mode |
| 17 | MGDG | DG(17:0/17:0) | 98896-81-2 | Cayman | Positive ion mode |
| 18 | DGDG | DG(17:0/17:0) | 98896-81-2 | Cayman | Positive ion mode |
| 19 | SQDG | DG(17:0/17:0) | 98896-81-2 | Cayman | Positive ion mode |
| 20 | DGTS | DG(17:0/17:0) | 98896-81-2 | Cayman | Positive ion mode |
| 21 | LDGTS | DG(17:0/17:0) | 98896-81-2 | Cayman | Positive ion mode |
| 22 | DGGA | DG(17:0/17:0) | 98896-81-2 | Cayman | Positive ion mode |
| 23 | ADGGA | DG(17:0/17:0) | 98896-81-2 | Cayman | Positive ion mode |
| 24 | DGCC | DG(17:0/17:0) | 98896-81-2 | Cayman | Positive ion mode |
| 25 | FFA | FFA(16:0)-d31 | 39756-30-4 | Supelco | negative ion mode |
| 26 | SPH | SPH(18:1)-d7 | 1246304-34-6 | Avanti | Positive ion mode |
| 27 | Cert | Cer(t18:0/22:0-d3) | 2011762-87-9 | Cayman | Positive ion mode |
| 28 | Cer | Cer(d18:1(d7)/18:0) | — | Avanti | Positive ion mode |
| 29 | HexCer | HexCer(d18:1(d5)/18:0) | 2260670-13-9 | Avanti | Positive ion mode |
| 30 | CE | CE(17:0) | 24365-37-5 | Rhawn | Positive ion mode |
| 31 | CoQ | CoQ10-d9 | 303-98-0 | Avanti | Positive ion mode |
